# SAR-Based Thermal Assessment of Dielectrophoretic Pulsed Electromagnetic Stimulation in Tibia Fractures with Metallic Implants

**DOI:** 10.3390/bioengineering13030364

**Published:** 2026-03-20

**Authors:** Abdullah Deniz Ertugrul, Erman Kibritoglu, Sinem Anil, Heba Yuksel

**Affiliations:** 1 Department of Electrical and Electronics Engineering, Bogazici University, Istanbul 34342, Turkey; abdullah.ertugrul@std.bogazici.edu.tr (A.D.E.); erman.kibritoglu@std.bogazici.edu.tr (E.K.); 2 Department of Physics, Bogazici University, Istanbul 34342, Turkey; sinem.anil@std.bogazici.edu.tr

**Keywords:** dielectrophoretic force, pulsed electromagnetic fields, orthopedic implants, specific absorption rate, implanted tibia fracture, electromagnetic heating, thermal safety, bioheat modeling

## Abstract

Electromagnetic field-based stimulation has emerged as a promising noninvasive approach for enhancing bone fracture healing. Beyond conventional pulsed electromagnetic field (PEMF) therapies employing spatially uniform fields, dielectrophoretic-force-based (DEPF) stimulation exploits electromagnetic field non-uniformities to induce localized interactions to enhance fracture healing. However, the thermal behavior associated with DEPF-driven PEMF exposure in the presence of metallic orthopedic implants remains largely unexplored. In this study, the thermal response of tissue-like tibia phantoms with and without metallic implants is investigated using an integrated experimental and numerical framework. A custom-designed conical coil is employed to generate non-uniform DEPF excitation capable of affecting the fracture site. Surface temperature evolution is measured using infrared thermal imaging, while electromagnetic power absorption is quantified through specific absorption rate (SAR)-based thermal measurement coupled with a bio-heat formulation. Anatomically realistic tibia phantoms reconstructed from computed tomography data are fabricated via a 3D printer to represent clinically relevant fracture configurations. Experimental results show that the metallic implant exhibits a rapid temperature increase of approximately 0.4 °C within the first few minutes of exposure, followed by thermal stabilization, corresponding to an effective absorbed power of ${\mathrm{SAR}}_{\mathrm{eff},\mathrm{implant}}\approx 2.2\;W/\mathrm{kg}$ inferred from the initial temperature slope. In contrast, the non-conductive resin phantom displays a temperature rise of only 0.05 °C over the same interval, yielding ${\mathrm{SAR}}_{\mathrm{eff},\mathrm{resin}}\approx 0.8\;W/\mathrm{kg}$. These findings demonstrate that implant-related eddy-current losses dominate localized heating under DEPF excitation, while tissue-like media remain weakly affected. This work provides SAR-based experimental evaluation of DEPF stimulation in implanted tibia fracture models, offering new insight into implant-induced electromagnetic heating and its implications for the safety and optimization of DEPF-based bone-healing therapies.

## 1. Introduction

Bone fractures represent a major clinical burden worldwide, with millions of cases reported annually and a substantial fraction progressing to delayed union or non-union [1,2,3]. Successful fracture healing depends on a complex interplay of mechanical stability, vascularization, cellular activity, and biochemical signaling [4]. Insufficient blood supply and impaired mass transport of oxygen and nutrients are widely recognized as key contributors to compromised healing outcomes, particularly in long bones such as the tibia [5,6,7].

Pulsed electromagnetic field (PEMF) therapy has been extensively investigated as a non-invasive adjunct treatment for enhancing bone regeneration and fracture healing [8,9,10,11,12]. A substantial body of experimental and clinical evidence demonstrates that PEMF exposure can modulate cellular behavior, stimulate osteogenic activity, and accelerate bone repair processes [13,14,15]. These therapeutic effects are generally attributed to electromagnetic interactions with electrically responsive structures at the cell membrane, whereby externally applied fields influence ion dynamics, membrane polarization, and associated intracellular signaling processes [9]. In particular, PEMF stimulation has been shown to alter calcium transport and membrane potential–dependent responses, leading to enhanced osteogenic differentiation and extracellular matrix synthesis. Moreover, PEMF exposure promotes the expression of key osteogenic and angiogenic factors, including bone morphogenetic protein-2 (BMP-2) and vascular endothelial growth factor (VEGF), which play central roles in coupling osteogenesis and vascularization during fracture repair [13,16]. Increased BMP-2 signaling supports osteoblast differentiation and bone matrix deposition, while upregulation of VEGF enhances angiogenesis and vascular permeability, thereby improving nutrient delivery and cellular recruitment in the fracture microenvironment. Such coordinated osteogenic and angiogenic responses support the clinical efficacy of PEMF therapy, which has been approved and adopted in several orthopedic applications, particularly for delayed and non-union fractures [17,18].

Despite extensive investigation of PEMF-mediated bone healing, prevailing mechanistic interpretations have largely focused on direct electromagnetic modulation of bone cells and their biochemical signaling. However, electromagnetic fields can also exert forces on charged or polarizable particles suspended in biological fluids, giving rise to electrophoretic and dielectrophoretic transport phenomena [19,20,21]. Such field-induced forces can influence the motion and spatial distribution of suspended constituents, including blood cells and macromolecules, thereby potentially modifying local mass transport, perfusion characteristics, and biochemical gradients in the fracture environment. Electrophoretic and dielectrophoretic manipulation of biological cells and particles are well established in bio-electromagnetics and microfluidic systems [22,23,24], yet its possible contribution to dielectrophoretic force (DEPF)-assisted bone regeneration has received comparatively limited attention. In particular, the hypothesis that electromagnetic stimulation may enhance fracture healing indirectly by altering blood-borne transport dynamics rather than solely through direct cellular stimulation remains largely unexplored in the orthopedic PEMF literature. This perspective motivates investigation of electromagnetic force–driven transport mechanisms in biologically relevant media, which forms the basis of the present study.

Beyond conventional PEMF approaches, recent studies have explored the role of DEPFs generated by spatially non-uniform electromagnetic fields [25]. Unlike uniform-field PEMF stimulation, DEPF-based approaches exploit field gradients to exert forces on polarizable particles, such as red blood cells and blood components, thereby offering a potential mechanism for enhancing local blood flow and mass transport within the fracture region. Several theoretical and numerical investigations have suggested that DEPF-based electromagnetic stimulation may provide additional control over blood flow enhancement compared to traditional PEMF systems [26].

Despite these advances, the existing literature exhibits two notable limitations. First, the majority of PEMF and DEPF studies focus on biological effects in homogeneous tissue models or in the absence of implanted metallic components. Second, thermal effects associated with electromagnetic exposure are often neglected or assessed only at a macroscopic level, without detailed consideration of localized power absorption mechanisms [27]. In particular, metallic orthopedic implants commonly used in tibial fracture stabilization are rarely incorporated into electromagnetic exposure models, even though their high electrical conductivity can significantly perturb local fields and induce eddy currents [28,29].

In contrast to prior studies, the present work provides a systematic evaluation of DEPF-based stimulation in the context of implanted tibial fractures, with explicit consideration of implant-induced electromagnetic interactions. This study presents a quantitative investigation of the thermal response of an implanted tibia configuration under DEPF-driven electromagnetic excitation using a specific absorption rate (SAR)–based framework. While existing PEMF studies largely assess biological efficacy without implants, and DEPF-related works predominantly focus on theoretical force generation or micro-fluidic applications, this study bridges these domains by experimentally and numerically evaluating SAR-driven heating effects in a realistic tissue–implant system. By doing so, the work directly addresses safety-relevant questions associated with implant-related eddy current formation and localized electromagnetic power deposition.

In this study, an integrated theoretical–experimental framework is developed to investigate electromagnetic exposure in bone-like samples with and without metallic implants. A non-uniform electromagnetic field is generated using a conical coil specifically designed to produce DEPF. The resulting electromagnetic power absorption is quantified using SAR analysis, and the associated thermal response is evaluated through controlled experiments and bio-heat numerical modeling. Anatomically realistic tibia phantoms reconstructed from computed tomography data are employed to replicate clinically relevant SAR scenarios.

The findings of this work provide new insight into the role of metallic implants in modulating electromagnetic field distributions, SAR localization, and thermal behavior under DEPF-based stimulation. By explicitly accounting for implant-induced field perturbations, this study contributes to a more comprehensive understanding of the safety and optimization of electromagnetic therapies for bone fracture healing and establishes a foundation for future in vivo and clinical investigations.

## 2. Theoretical Framework

In bio-electromagnetic exposure analysis, the SAR is the standard dosimetric quantity used to quantify the rate at which electromagnetic energy is absorbed by biological tissue [30,31]. SAR represents the absorbed electromagnetic power per unit mass and provides a physically significant relation between the applied electromagnetic fields and the resulting thermal effects in tissue [31].

For non-uniform electric fields and spatially varying electrical properties, the local SAR distribution is defined as

(1) $$\mathrm{SAR}\left(r\right)=\frac{\sigma (r)}{\rho (r)}{\left|E_{\mathrm{rms}}\left(r\right)\right|}^{2},$$

where σ(r) denotes the electrical conductivity of the tissue (S/m), ρ(r) is the tissue density (kg/m^3^), and $E_{\mathrm{rms}}\left(r\right)$ is the root-mean-square (RMS) value of the induced electric field (V/m). This formulation enables pointwise evaluation of electromagnetic energy absorption under spatially heterogeneous conditions and is consistent with established bioelectromagnetic dosimetry standards [32,33].

The SAR distribution serves as a compact representation of electromagnetic heating, provided that the RMS electric field is evaluated for the applied excitation waveform. Consequently, SAR is employed as the primary coupling variable between the electromagnetic and thermal models in this study.

The temperature evolution within biological tissue subjected to electromagnetic exposure is governed by heat conduction, perfusion-mediated heat transfer, metabolic heat generation, and external energy deposition [34]. These mechanisms are commonly described using Pennes’ bioheat equation, which is expressed as

(2) $$\rho c\frac{\partial T}{\partial t}=\nabla \cdot \left(k\nabla T\right)+\rho_{b}c_{b}\omega_{b}\left(T_{b}-T\right)+Q_{\mathrm{met}}+Q_{\mathrm{ext}},$$

where *T* denotes the tissue temperature (K), ρ is the tissue density (kg/m^3^), *c* is the specific heat capacity (J/kg·K), and *k* is the thermal conductivity (W/m·K) [35,36]. The parameters $\rho_{b}$, $c_{b}$, and $\omega_{b}$ represent the density, specific heat capacity, and perfusion rate of blood, respectively, while $T_{b}$ is the arterial blood temperature. The terms $Q_{\mathrm{met}}$ and $Q_{\mathrm{ext}}$ correspond to metabolic heat generation and externally applied heat sources.

In the present framework, electromagnetic heating is incorporated into the thermal model through the SAR formulation. Accordingly, the external heat source term is defined as

(3) $$Q_{\mathrm{ext}}=\rho \;\mathrm{SAR}\left(r\right),$$

which establishes a direct and physically consistent coupling between electromagnetic power absorption and temperature rise.

Substituting this expression into Equation (2), the governing equation becomes

(4) $$\rho c\frac{\partial T}{\partial t}=\nabla \cdot \left(k\nabla T\right)+\rho_{b}c_{b}\omega_{b}\left(T_{b}-T\right)+Q_{\mathrm{met}}+\rho \;\mathrm{SAR}\left(r\right).$$

For the experimental conditions considered in this study, metabolic heat generation and blood perfusion effects are absent. Therefore, the bioheat equation reduces to

(5) $$\rho c\frac{\partial T}{\partial t}=k\nabla^{2}T+\rho \;\mathrm{SAR}\left(r\right),$$

which describes transient heat diffusion driven only by electromagnetic energy absorption.

This reduced formulation allows the thermal response induced by the applied electromagnetic fields to be isolated and quantitatively assessed. This makes it particularly suitable for controlled numerical simulations and experimental validation.

## 3. Materials and Methods

This study employs an integrated numerical and experimental methodology to investigate the thermal effects induced by DEPF stimulation in bone-like samples, with particular emphasis on tissues in the vicinity of metallic orthopedic implants. The combined approach enables both controlled experimental observation and theoretical interpretation of electromagnetic heating mechanisms in conductive biological environments.

The experimental component focuses on the generation of well-defined electromagnetic fields using a custom-designed coil system. The measurement of the resulting temperature distributions are taken via non-contact thermal imaging. Similarly, the theoretical part of the study relies on computational modeling based on SAR analysis and Pennes’ bio-heat equation in order to quantify the electromagnetic power deposition and the associated thermal response.

By integrating experimental measurements with numerical modeling, the proposed methodology allows for the systematic evaluation of electromagnetic exposure conditions and their thermal effects, particularly in the presence of metallic implants, where field enhancement and localized heating may occur.

### 3.1. Coil Geometry and Parametrization

The coil comprised N=40 turns of copper wire (cross-sectional area $0.82\;{\mathrm{mm}}^{2}$, AWG 18), wound on a non-magnetic former. The coil was driven using a PWM current waveform with repetition rate f=10 kHz and duty cycle D=50%. The applied current was $I_{\mathrm{rms}}=0.75\;A$ (measured at the coil terminals), and this RMS value was used as the excitation input for the numerical electromagnetic model. The exposure duration was 60 min, and the coil-to-sample positioning was fixed using a dedicated non-conductive holder to ensure reproducible alignment between the coil and the tibia phantom.

**Figure 1:**
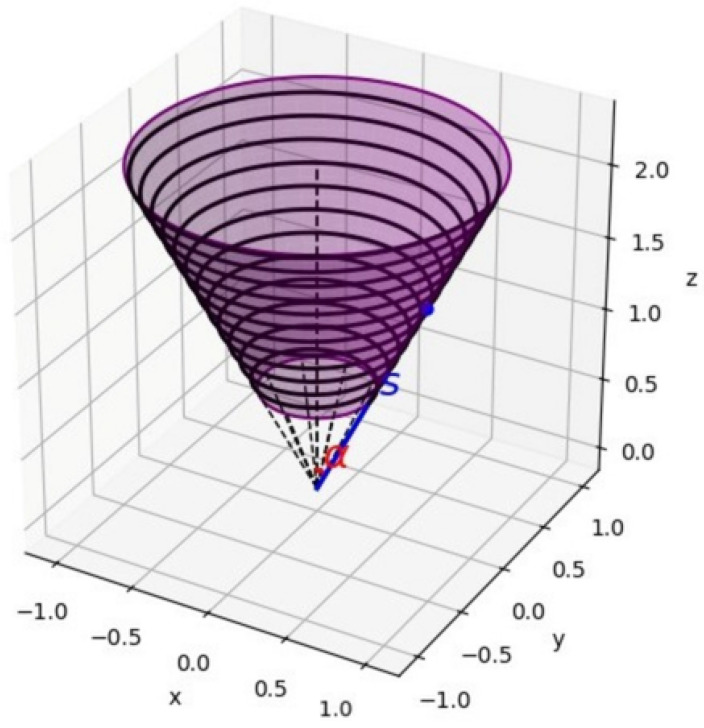
Schematic illustration of the uniformly wound conical coil geometry. The half-angle α defines the inclination of the cone surface relative to the symmetry axis.

Electromagnetic excitation is generated using a uniformly wound conical coil. Figure 1 illustrates the geometric configuration of the winding, characterized by a half-angle α between the cone axis and its generatrix.

The coil is wound over a finite frustum section of the cone, bounded by the slant-coordinate limits $s_{1}$ and $s_{2}$. The effective slant length of the winding region is therefore

(6) $$L=s_{2}-s_{1}.$$

A uniformly wound conductor follows a conical helical path parameterized by the azimuthal angle ϕ, which increases linearly with the slant coordinate *s*:

(7) ϕ(s)=ks,

where the angular winding rate is defined as

(8) $$k=\frac{2\pi N}{L},$$

with *N* denoting the total number of turns uniformly distributed along the slant length.

For a cone with half-angle α, the radial and axial coordinates of a point on the conical surface are given by

(9) r(s)=ssinα,  z(s)=scosα.

Accordingly, the parametric representation of the helical winding is expressed as

(10) $$r\left(s\right)=\left(s\sin\alpha \cos(ks),\;s\sin\alpha \sin(ks),\;s\cos\alpha \right).$$

### 3.2. Magnetic Flux Density Formulation

The magnetic flux density generated by the conical winding is derived using the Biot–Savart law [37,38]. To obtain physically interpretable expressions while preserving analytical tractability, the formulation is evaluated for observation points located on the symmetry axis of the coil.

For an observation point at $z=z_{0}$ on the *z*-axis, the magnetic flux density components are

(11) $$\begin{array}{cc} B_{x}\left(z_{0}\right) & =\frac{\mu_{0}I}{4\pi }\int_{s_{1}}^{s_{2}}\frac{C_{x}\left(s\right)}{{\left(s^{2}-2sz_{0}\cos\alpha +z_{0}^{2}\right)}^{3/2}}\;ds, \end{array}$$

(12) $$\begin{array}{cc} B_{y}\left(z_{0}\right) & =\frac{\mu_{0}I}{4\pi }\int_{s_{1}}^{s_{2}}\frac{C_{y}\left(s\right)}{{\left(s^{2}-2sz_{0}\cos\alpha +z_{0}^{2}\right)}^{3/2}}\;ds, \end{array}$$

(13) $$\begin{array}{cc} B_{z}\left(z_{0}\right) & =\frac{\mu_{0}I}{4\pi }\int_{s_{1}}^{s_{2}}\frac{C_{z}\left(s\right)}{{\left(s^{2}-2sz_{0}\cos\alpha +z_{0}^{2}\right)}^{3/2}}\;ds, \end{array}$$

where the auxiliary functions are defined as

(14) $$\begin{array}{cc} C_{x}\left(s\right) & ≜\sin\left(\alpha \right)\left[-ks^{2}\cos\left(\alpha \right)\cos\left(ks\right)+ksz_{0}\cos\left(ks\right)+z_{0}\sin\left(ks\right)\right], \end{array}$$

(15) $$\begin{array}{cc} C_{y}\left(s\right) & ≜\sin\left(\alpha \right)\left[-ks^{2}\cos\left(\alpha \right)\sin\left(ks\right)+ksz_{0}\sin\left(ks\right)-z_{0}\cos\left(ks\right)\right], \end{array}$$

(16) $$\begin{array}{cc} C_{z}\left(s\right) & ≜ks^{2}\sin^{2}\left(\alpha \right).\;\;\;\;\;\;\;\;\;\;\;\;\;\;\;\;\;\; \end{array}$$

Although these expressions are analytically defined, they do not reduce to closed-form solutions and are therefore evaluated numerically. Numerical integration is performed for different winding densities *N* and cone half-angles α to systematically investigate the influence of coil geometry on the axial magnetic field distribution.

**Figure 2:**
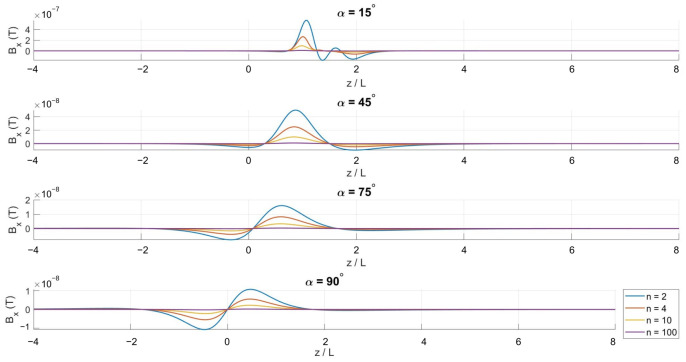
Axial variation in the *x*-component of the magnetic flux density for $\alpha =15^{\circ }$, $45^{\circ }$, $75^{\circ }$, and $90^{\circ }$.

**Figure 3:**
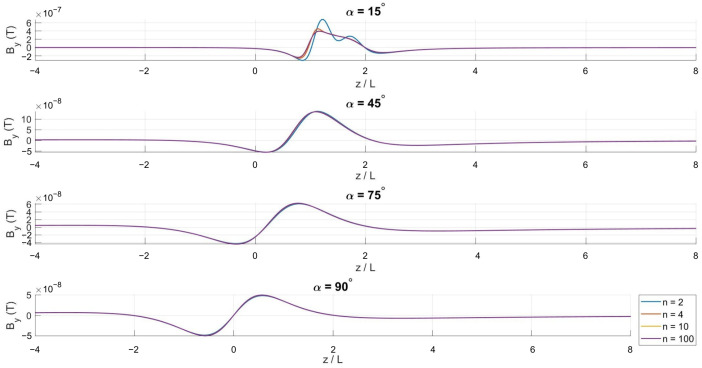
Axial variation in the *y*-component of the magnetic flux density for $\alpha =15^{\circ }$, $45^{\circ }$, $75^{\circ }$, and $90^{\circ }$.

**Figure 4:**
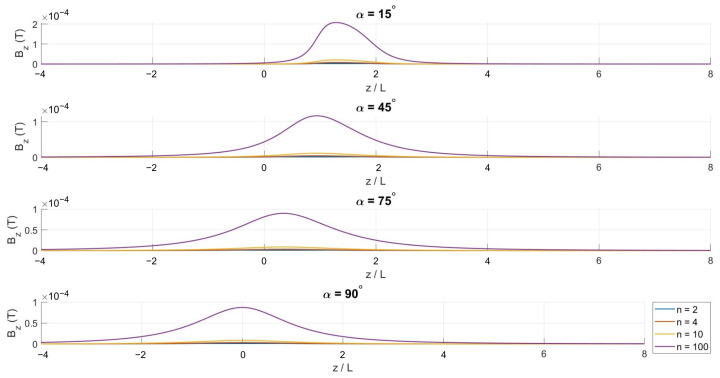
Axial variation in the dominant *z*-component of the magnetic flux density for $\alpha =15^{\circ }$, $45^{\circ }$, $75^{\circ }$, and $90^{\circ }$.

Figure 2, Figure 3 and Figure 4 show the spatial variation in the magnetic flux density components along the symmetry axis. The axial component $B_{z}$ remains dominant across all configurations, reflecting the inherent symmetry of the conical winding.

The magnetic flux density distribution was computed using a quasi-static electromagnetic model of the full coil phantom implant configuration. Within the fracture site region of interest (ROI) in the tibia model, the predicted magnetic flux density magnitude ranged from approximately 0.05 mT to 0.2 mT (Figure 4), demonstrating a spatially non-uniform field distribution across the bone volume. These levels fall within the lower range of magnetic flux densities commonly reported in PEMF and inductive bone-stimulation studies, which typically span sub-millitesla to millitesla levels (approximately 0.1–10 mT) depending on coil geometry and excitation amplitude [39,40,41,42]. Such magnitudes are consistent with coil excitations on the order of 0.5–1 A in laboratory and clinical PEMF applicators.

### 3.3. Three-Dimensional Field Visualization

**Figure 5:**
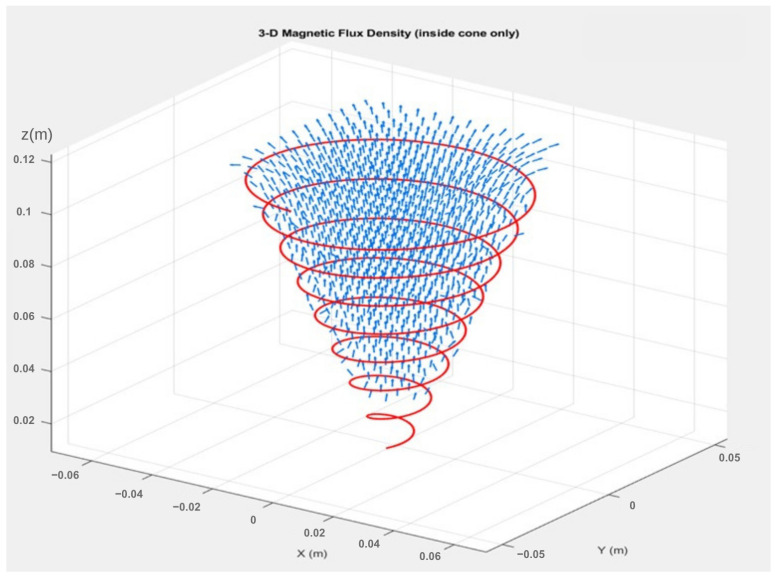
Qualitative three-dimensional visualization of the magnetic flux density vectors inside the conical coil. The helical winding is shown in red, and the magnetic field vectors are shown in blue.

To illustrate the magnetic field distribution inside the coil volume, the formulation is extended numerically to the full three-dimensional interior of the cone. The resulting magnetic flux density B(x,y,z) is visualized in Figure 5 using a three-dimensional quiver representation.

### 3.4. Thermal Simulation Configurations

When a time-varying magnetic field is applied to conductive materials, eddy currents are induced according to Faraday’s law of electromagnetic induction [29]. While induced currents in biological tissues are typically weak due to low conductivity, the presence of metallic implants can lead to substantial eddy current formation and associated ohmic losses [28,37].

**Figure 6:**
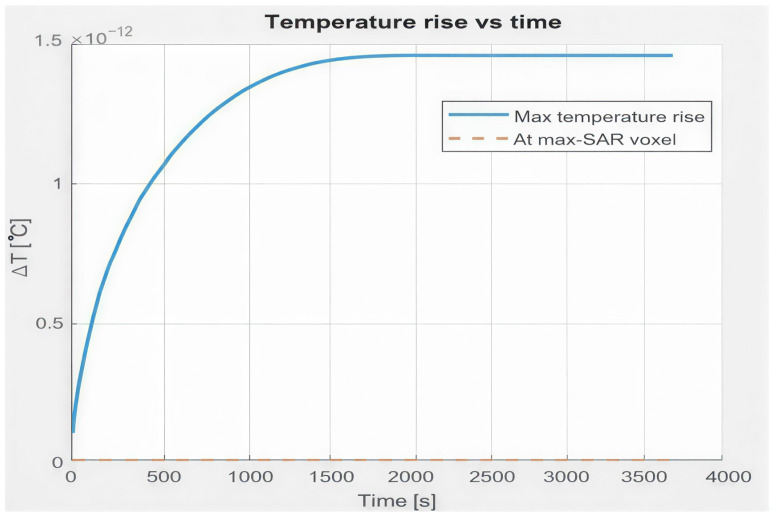
Temporal evolution of temperature rise for the tissue-like medium, showing negligible heating under electromagnetic exposure.

The resulting temperature field T(r,t) is evaluated for two configurations. In the first configuration, the region inside the coil is modeled as a homogeneous tissue-like medium. The predicted temperature rise remains extremely small, in the order of $10^{-12}$, as shown in Figure 6, indicating negligible thermal effects.

**Figure 7:**
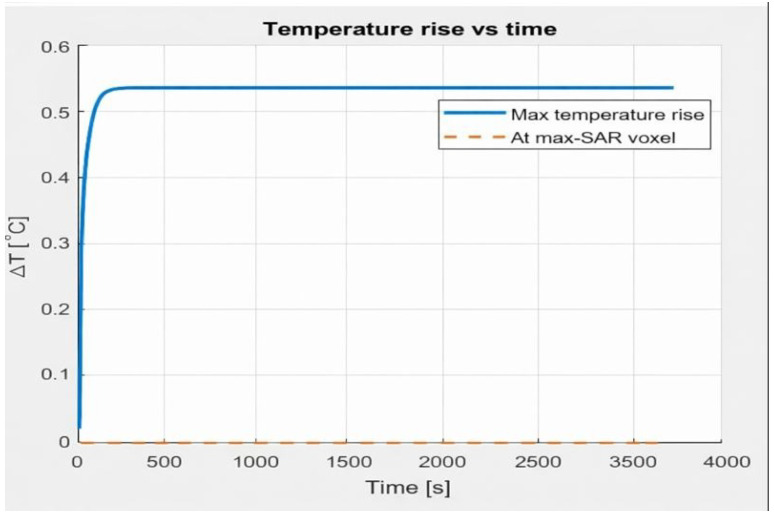
Temporal evolution of temperature rise for the metallic medium, illustrating enhanced electromagnetic heating due to induced eddy currents.

In contrast, when a metallic cone made of steel is placed inside the coil, the model predicts a pronounced temperature increase, reaching approximately 0.5 °C within the first minutes before approaching steady-state conditions. The corresponding temperature evolution is shown in Figure 7.

### 3.5. Tibia Phantom Reconstruction and Fabrication

Given the widespread clinical use of metallic implants in tibial fracture stabilization, the human tibia is selected as the representative anatomical structure [43,44]. To replicate biologically relevant conditions, anatomically accurate tibia phantoms are constructed using CT-based reconstruction and additive manufacturing. The tibial geometry is extracted from computed tomography data using the open-source, software 3D Slicer version 5.10.0, segmented via thresholding and manual refinement, and exported as an STL file for fabrication. The phantom is manufactured using PLA filament to approximate the structural and dielectric characteristics of cortical bone.

To enable anatomically realistic experimental and numerical investigations, a subject-specific tibia phantom was reconstructed from computed tomography (CT) imaging data and fabricated using high-resolution additive manufacturing techniques.

The human tibia was selected as the anatomical structure of interest due to its well-defined morphology and relevance for lower-limb bio-mechanical modeling. To ensure anatomically realistic representation, a subject-specific tibia phantom was reconstructed from CT imaging data and subsequently fabricated using high-resolution additive manufacturing techniques.

### 3.6. Data Import and Initial Segmentation

**Figure 8:**
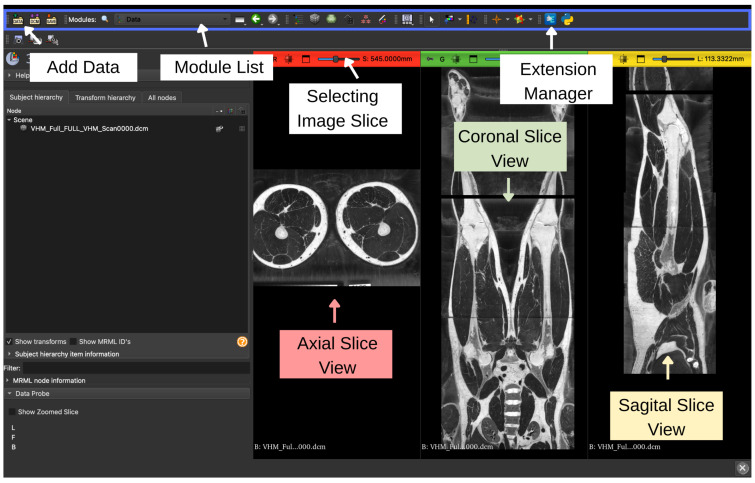
Overview of the 3D Slicer interface. The top toolbar provides access to data loading (Add Data), module selection (Module List), slice selection controls, and the Extension Manager. The left panel displays the Subject Hierarchy and module-specific user interface. The main viewing area shows synchronized orthogonal slice views of the loaded DICOM dataset: axial (left), coronal (center), and sagittal (right).

The CT dataset [45], provided in Digital Imaging and Communications in Medicine (DICOM) format, was imported into 3D Slicer version 5.10.0 using the *DICOM* module. After loading, the orientation and anatomical alignment of the volume were verified in axial, sagittal, and coronal slice views to ensure the correct visualization of the tibia. A new segmentation was then created using the Segment Editor module. The imported CT volume and the initialized segmentation environment are shown in Figure 8.

### 3.7. Threshold-Based Bone Segmentation

Initial segmentation of osseous structures was performed using intensity thresholding. In CT imaging, the cortical bone exhibits significantly higher Hounsfield Unit (HU) values than the surrounding soft tissues, typically exceeding +200 HU and reaching values on the order of +1000 HU or higher. Accordingly, the *Threshold* effect in 3D Slicer was applied with a lower threshold of approximately 200 HU, while the upper threshold was set to the maximum intensity present in the scan [46].

**Figure 9:**
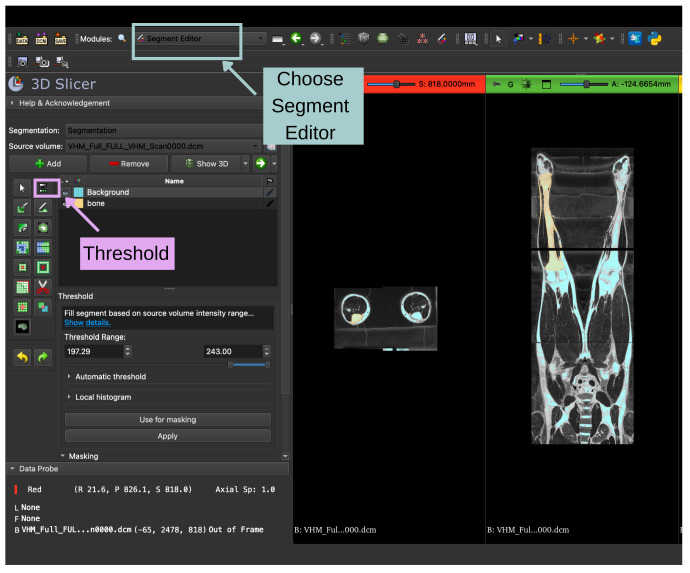
Segmentation workflow in 3D Slicer using the Segment Editor module. After selecting the source DICOM volume, a new segment (“bone”) is created and the Threshold tool is applied to isolate high-intensity structures based on a defined grayscale intensity range (197–243).

This thresholding operation automatically selected all high-density structures within the volume, corresponding to the skeletal system. Threshold values were refined using histogram inspection and slice-by-slice evaluation to ensure complete inclusion of the tibial cortex while minimizing non-bone regions. The tools used in the *Segment Editor Module* are shown in Figure 9.

### 3.8. Isolation of the Tibia

**Figure 10:**
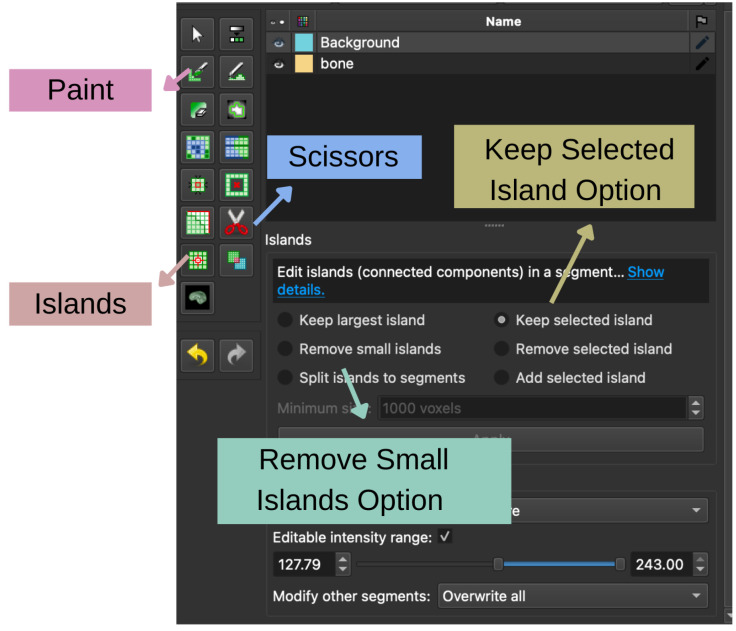
Manual refinement tools in the Segment Editor module of 3D Slicer.

After thresholding, the segmentation included multiple skeletal structures. The tibia was isolated using others tools available in the *Segment Editor* module shown in Figure 10.

Firstly, the *Islands* effect was used. The *Keep Selected Island* option (Figure 10) allowed selection of a voxel inside the tibia in slice or 3D view, keeping only the tibia and removing other disconnected regions.

Then, manual refinement was performed using the *Erase* and *Scissors* tools (Figure 10) while examining axial, coronal, and sagittal slice views. The removal of surrounding bones was guided by anatomical criteria based on *Netter’s Atlas of Human Anatomy* [47].

This guide includes the following:

- The tibial plateau at the proximal end;
- The continuous cortical structure of the tibial shaft;
- The distal tibial plafond and medial malleolus.

Voxels were removed only when their location and shape were not consistent with tibial anatomy according to the atlas. All edits were performed slice-by-slice and checked in orthogonal planes to preserve cortical integrity and avoid over-segmentation.

This approach is consistent with previously reported semi-automatic bone segmentation methods [48].

After refinement, all slices were reviewed to ensure that (i) the complete tibia was preserved, (ii) cortical continuity was maintained, and (iii) no remaining fragments from neighboring bones were present.

### 3.9. Segmentation Validation and Post-Processing

A three-dimensional surface representation of the segmented tibia was generated by enabling the *Show 3D* option in the *Segment Editor*. This visualization allowed confirmation that the tibia was the only remaining structure and that its geometry was continuous.

Residual artifacts, such as small isolated fragments or noise voxels, were removed using the *Remove Small Islands* function shown in Figure 10. Mild surface smoothing was applied using the *Smoothing* effect to reduce voxelization artifacts and improve geometric quality.

### 3.10. Labelmap Export and Masked Volume Generation

The finalized tibia segmentation was exported as a binary labelmap volume, in which voxels are assigned integer labels (1 for tibia and 0 for background), using the *Export visible segments to binary labelmap* option in the *Segmentations* module.

Using this labelmap, a masked CT volume containing only the tibia was generated with the *Mask Scalar Volume* module. Voxels corresponding to the tibia retained their original HU values, while all other voxels were set to zero. The masked volume enabled isolated visualization of the tibia while preserving grayscale intensity information for validation.

### 3.11. Three-Dimensional Rendering and Model Preparation

**Figure 11:**
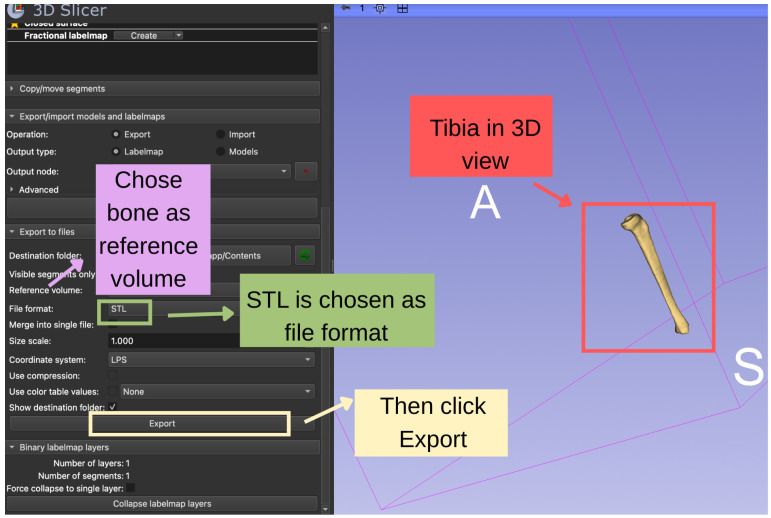
Exportation steps of the segmented tibia in 3D Slicer. The bone segment was selected as the reference volume and saved in STL format using the Export/Import Models and Labelmaps panel. After clicking Export, the tibia was generated and displayed in the 3D view.

The surface model of the tibia was finally exported in STL format in *Segmentations Module*, as shown in Figure 11.

### 3.12. Manufacturing of the Tibia Phantom

**Figure 12:**
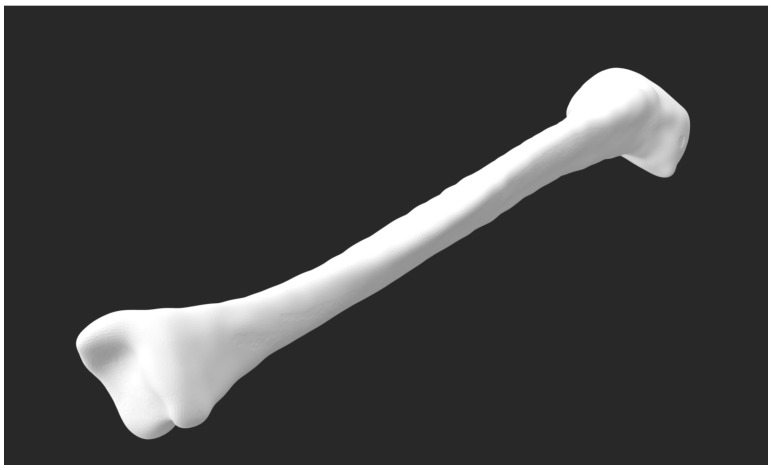
Three-dimensional surface model of the tibia exported in STL format.

The finalized STL format prepared is shown in Figure 12 for fabrication using a high-resolution masked stereolithography (MSLA) additive manufacturing system. Fabrication was performed using a Phrozen Mega 8K S 3D printer (Hsinchu, Taiwan), which provides ultra-high spatial resolution suitable for reproducing fine anatomical features of tibial bone geometry.

Prior to printing, the STL file was imported into the printer’s slicing software and oriented to minimize support contact with anatomically relevant surfaces and to reduce layer-induced stair-stepping effects along the tibial shaft. Support structures were automatically generated and manually refined to ensure mechanical stability during fabrication while preserving surface fidelity in regions of interest.

The tibia phantom was fabricated using a standard photopolymer resin compatible with MSLA printing. Printing parameters were selected to balance dimensional accuracy, surface quality, and structural integrity. A summary of the key printer settings used in this study is provided in Table 1.

Following fabrication, the printed tibia phantom was cleaned in isopropyl alcohol to remove uncured resin and subsequently post-cured under ultraviolet illumination to ensure complete polymerization. Support structures were carefully removed, and the surface was visually inspected to verify geometric integrity and absence of fabrication defects.

The resulting tibia phantom accurately reproduced the external morphology of the segmented bone and provided a high-fidelity physical representation suitable for subsequent experimental and numerical investigations requiring anatomically realistic geometry.

### 3.13. Experimental Protocol and Measurement Procedure

The experiment is run on two different configurations: the non-implant model, representing intact bone tissue, and the implant model, which is a metallic implant used to analyze implant-related field perturbations. These configurations allow direct comparisons of temperature between human tissues and metallic implants.

In both configurations, the sample is placed concentrically to ensure a symmetrically distributed electromagnetic field distribution. In each trial, the sample is subjected to the coil-generated electromagnetic field for periods ranging from 1 min to 1 h under controlled environmental temperature conditions. In each trial, field parameters such as voltage, current, frequency, duty cycle, etc., are kept constant. Current and voltage values are recorded throughout each trial to ensure experimental reproducibility. Between successive runs, the system is allowed to return to environmental temperature to eliminate residual thermal effects from previous trials.

In each of the measurements, temperature readings are extracted from the same predefined regions to ensure the spatial coherence of the measurements. These predefined regions correspond to points where the applied electromagnetic field is expected to be maximum based on the theoretical results. To reduce external thermal interference, all measurements are taken while the sample is placed on a styrofoam insulation platform to further provide thermal isolation from the underlying surface. Furthermore, the measurements are taken from a fixed distance from the sample in every trial to prevent distance-related perturbations in the recordings.

The surface temperature was measured using an infrared thermography camera (Bosch GTC 400, Bosch GmbH, Gerlingen, Germany). The camera has a stated thermal sensitivity of approximately 0.05 °C. The emissivity was set according to the phantom surface material and verified using a reference rod of identical surface finish positioned outside the coil and allowed to reach thermal equilibrium with the phantom prior to exposure. The camera was mounted at a fixed distance and viewing angle to minimize geometric variation and specular reflections; the phantom surface was resin and no reflective metallic structures were present in the field of view.

The ambient laboratory temperature during measurements was maintained at 23±1 °C with negligible airflow. The phantom and the external reference rod were allowed to thermally equilibrate with the ambient environment for at least 30 min prior to stimulation. The initial phantom surface temperature was confirmed to be spatially uniform within ±0.05 °C before each trial.

Reported temperature changes were derived from time-resolved temperature profiles averaged over a fixed region of interest on the implant or phantom surface. Repeat measurements (n=10) showed consistent temperature evolution within the camera noise level. Considering camera accuracy, thermal sensitivity, and repeatability, the uncertainty in the measured temperature rise was estimated to be approximately ±0.05 °C. Error bars reflecting this uncertainty have been added to the reported peak temperature increases of approximately 0.4 °C (implant) and 0.05 °C (resin phantom).

To ensure rigorous assessment of the thermal behavior induced by the DEPF, the measurements are subjected to a systematic statistical analysis. For each different measurement conditions, such as the frequency and the amplitude of the applied input signal and the duration temperature, measurements are collected at discrete points. Starting from the initial temperature, measurements are taken at the end of ten-minute intervals. For each measurement, the values are converted to the temperature changes by subtracting the initial values from each measurement.

For every group of repeated trials, the mean is calculated to represent the typical behavior of the thermal response of the sample. Moreover, the standard deviations for each repeated trial with different parameters are calculated to provide a clearer picture of how consistently each sample responds to the applied field.

### 3.14. Experimental Setup

A conical coil is designed and fabricated to generate a spatially non-uniform magnetic field within the region of interest [49]. The coil is 3D-printed to ensure geometric accuracy and mechanical stability, and a copper wire was uniformly wound according to the coil design principles. The bone sample is positioned along the symmetry axis of the coil, where the magnetic field distribution is most clearly defined. Surface temperature variations in the sample are monitored using a non-contact infrared thermal imaging camera.

**Figure 13:**
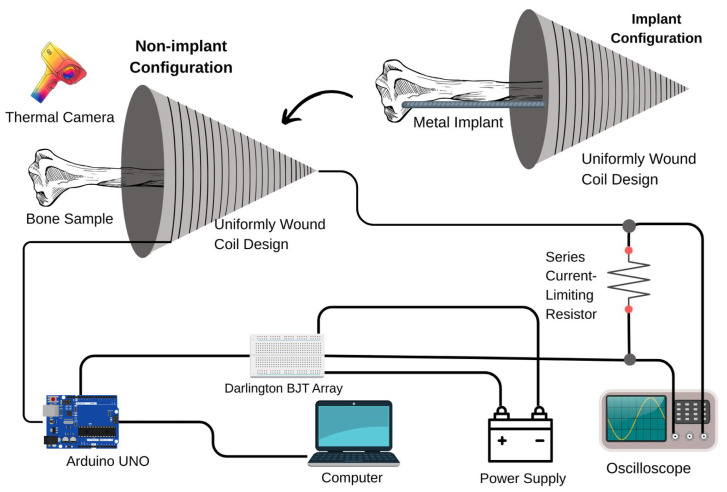
Schematic representation of the experimental setup, including the conical coil, excitation circuitry, control unit, and thermal imaging system.

**Figure 14:**
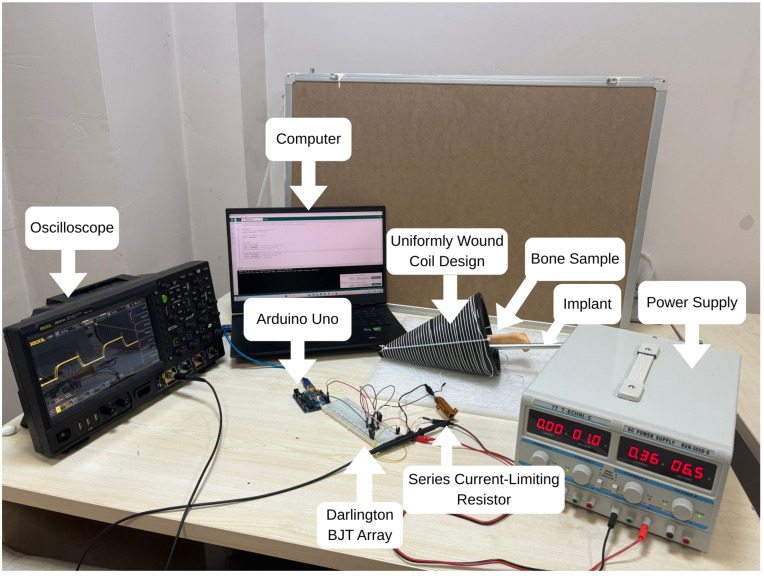
Real picture of the experimental setup showing the uniformly wound conical coil, bone sample, excitation and control circuitry.

The electromagnetic excitation circuit consisted of an external power supply, a series current-limiting resistor, an Arduino-based control unit implemented on a breadboard, and a computer used to generate square-wave control signals. The series resistor is included as a safety measure to limit the maximum current flowing through the coil. A schematic representation of the experimental setup is shown in Figure 13. Additionally, the real setup is shown in Figure 14.

The coil is driven using an Arduino-UNO-controlled Darlington BJT array (ULN2003) (Texas Instruments, Dallas, TX, USA), capable of generating square-wave excitation signals within a tunable frequency range of 1 Hz–20 kHz. The current amplitude is adjusted to regulate the resulting magnetic flux density. The ULN2003 array serves as the power interface between the microcontroller and the inductive load. Each channel of the array consists of two cascaded NPN transistors in a Darlington configuration, enabling current levels of up to approximately 800 mA while requiring only a few milliamperes at the microcontroller input pins.

When the digital control signal is high, the Darlington pair saturates and provides a low-impedance conduction path for the coil current [50]. When the signal is low, the transistors switch off and interrupt current flow. Due to the inductive nature of the coil and the square-modulated excitation waveform, rapid switching transitions generate reverse-voltage spikes [51]. To protect the switching elements, each channel of the ULN2003 array includes an internal flyback diode connected between the output node and the common supply rail. This diode provides a safe recirculation path for the stored magnetic energy during current turn-off, preventing damage to the transistors and enabling stable operation at higher switching frequencies.

**Figure 15:**
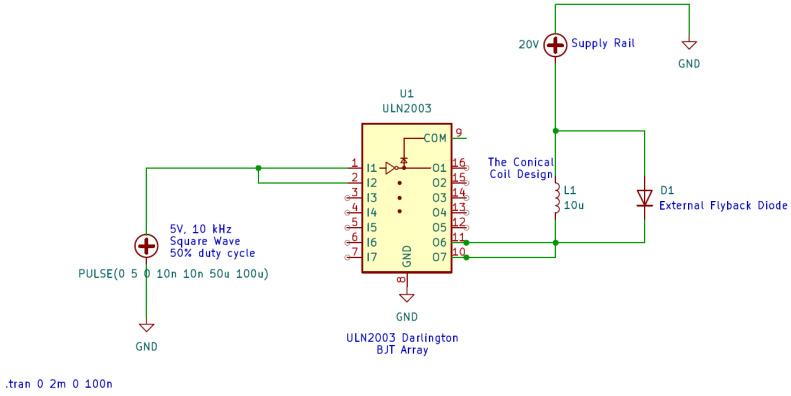
ULN2003-based switching circuit used to drive the inductive coil.

**Figure 16:**
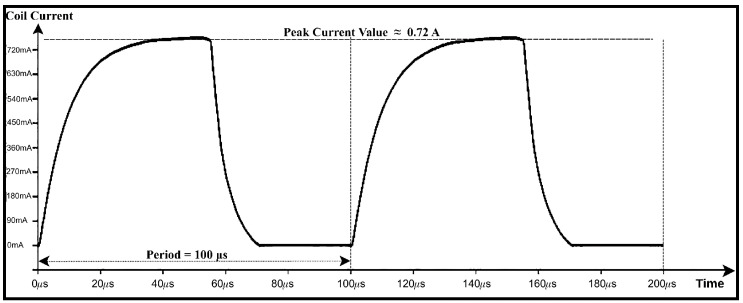
LTspice simulation results of the ULN2003-based switching circuit under a 5 V square-wave control signal, illustrating inductive current behavior and voltage clamping.

To validate the behavior of the switching stage under inductive loading, the ULN2003-based circuit is modeled in LTspice using a 20 V supply and a 10 μH inductance representing the coil. A 5 V square-wave control signal is applied to the input of the Darlington array. The simulated coil current exhibits the characteristic exponential rise and decay expected from an RL network. Peak current level of 0.72 A is obtained, and the reverse-voltage transients generated during turn-off are effectively clamped by the internal flyback diodes. The simulated switching circuit and corresponding results are shown in Figure 15 and Figure 16, respectively.

The configuration shown in Figure 15 enables the controlled square-wave excitation of the conical coil through a dedicated switching stage. The circuit exhibits how the excitation current flowing through the coil is generated and controlled. The induced magnetic field strength, spatial distribution, and resulting eddy current formation directly depend on the magnitude, waveform, and stability of the driving current. The use of the ULN2003 Darlington array ensures stable switching of the inductive load while maintaining a constant excitation frequency. The inclusion of flyback protection prevents voltage transients during turn-off. Therefore, this configuration provides a well-defined excitation waveform for the coil throughout the experiments while maintaining constant switching frequency.

## 4. Results

**Figure 17:**
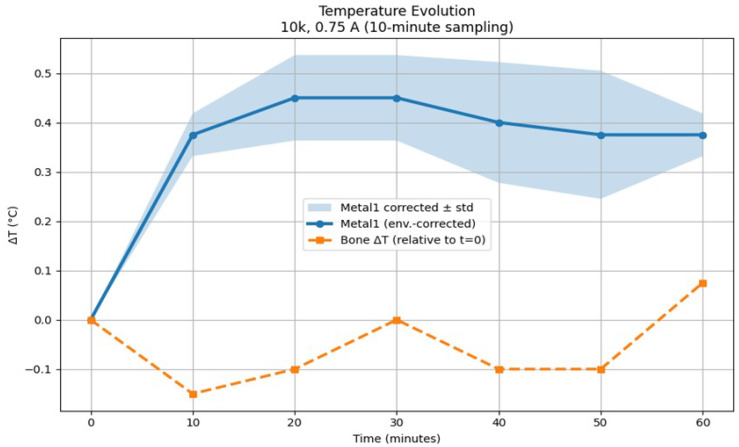
Temporal evolution of temperature for implant and non-implant configurations under a 10 kHz, 0.75 A electromagnetic excitation. Shaded regions indicate the standard deviation of repeated measurements.

Figure 17 presents the temporal evolution of surface temperature for the implant and non-implant configurations under a square-wave excitation with a frequency of 10 kHz and a current amplitude of 0.75 A. Temperature measurements are acquired at 10 min intervals over a total exposure duration of 60 min. To account for ambient temperature variations, the temperature of the metallic implant (Metal1) is environmentally corrected using a reference metallic sample (Metal2), which was not exposed to the electromagnetic field.

The 0–20 min interval was selected based on both the measured temperature evolution and the short-time behavior of volumetric heating. During the initial heating phase, the temperature rise exhibited an approximately linear increase, consistent with the short-time limit of the bio-heat equation in which conductive diffusion and boundary heat losses remain small relative to volumetric power deposition. Beyond approximately 20 min, the temperature curve showed a clear reduction in slope and onset of thermal stabilization, indicating increasing influence of conductive heat spreading within the phantom and convective and radiative heat exchange with the surrounding air. Accordingly, only the initial 0–20 min interval was used for ${\mathrm{SAR}}_{\mathrm{eff}}$ estimation to ensure validity of the linear temperature-rise assumption.

As shown in Figure 17, the implant configuration exhibits a rapid temperature increase following the onset of excitation. Within the first 10 min, the environmentally corrected temperature rise of the implant reaches approximately 0.4 °C. The temperature continues to increase slightly, reaching a maximum of approximately 0.45 °C after 20–30 min, after which a quasi-steady-state behavior is observed. Beyond this point, only minor fluctuations are present, as reflected by the relatively narrow standard deviation band.

Thermal stabilization was defined quantitatively as the time at which the absolute temperature rise rate satisfies $|dT/dt|<1\;\times \;10^{-4}\;K\;s^{-1}$ over a continuous 10 min interval. Using this criterion, the implant temperature stabilized rapidly, and the maximum observed surface temperature rise over the full 60 min exposure was $\Delta T_{\max}\approx 0.45\;{}^{\circ}C$.

In contrast, the non-implant configuration remains close to its initial temperature throughout the measurement period. The observed temperature variation remains centered around 0 °C, with deviations not exceeding approximately 0.1 °C. This behavior indicates that no measurable thermal response occurs in the absence of a metallic implant under 0.75 A excitation at 10 kHz.

**Figure 18:**
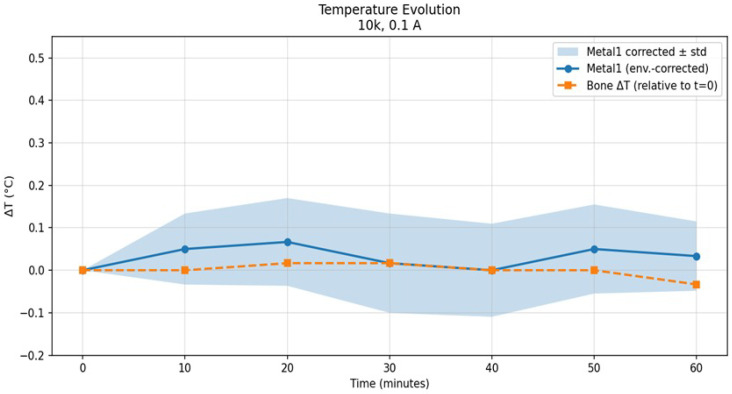
Temporal evolution of temperature for implant and non-implant configurations under a 10 kHz, 0.1 A electromagnetic excitation. Shaded regions indicate the standard deviation of repeated measurements.

Figure 18 shows the corresponding temperature evolution for both configurations under the same excitation frequency of 10 kHz but with a reduced current amplitude of 0.1 A.

At the lower current amplitude, the temperature response of the implant configuration is substantially reduced. The environmentally corrected mean temperature change remains below 0.1 °C throughout the exposure period and does not exhibit a clear monotonic increase with time. Similarly, the non-implant configuration remains close to the baseline temperature, showing only small fluctuations within the measurement uncertainty.

Table 2 and Table 3 summarize the temporal evolution of temperature variations measured at 10 kHz for two current amplitudes (0.75 A and 0.1 A), with values reported as mean ± standard deviation over ten repeated measurements. For the higher current condition (0.75 A), the metallic component exhibited a gradual temperature increase during the first 20–30 min, reaching a maximum mean temperature rise of approximately 0.45 °C.

In contrast, temperature variations measured within the bone region remained minimal throughout the experiment. Across all time points and for both current amplitudes, bone temperature changes were confined within ±0.1 °C relative to the initial baseline. For the lower current condition (0.1 A), both metallic and bone temperature variations remained close to baseline values, with no systematic increase observed over time.

The clear separation between the thermal responses of metallic and bone regions indicates that the applied electromagnetic excitation at 10 kHz primarily affects conductive components, while thermal diffusion into bone tissue remains negligible under the investigated conditions. These results demonstrate that, within the tested parameter range, the experimental protocol does not induce biologically relevant temperature elevations in bone.

The results demonstrate a clear dependence of the thermal response on both the presence of a metallic implant and the excitation current amplitude. While negligible temperature changes are observed for both configurations at low current amplitudes, higher current excitation leads to a pronounced and reproducible temperature rise exclusively in the implant configuration. This contrast highlights the role of metallic implants in modulating electromagnetic field-induced heating under DEPF exposure.

The tibia phantom was fabricated from a photo-polymer resin with electromagnetic and thermal properties representative of common 3D-printing materials: electrical conductivity $\sigma_{\mathrm{resin}}\approx 1\times 10^{-8}\;S\;m^{-1}$, relative permittivity $\varepsilon_{r}\approx 2.5$, and relative permeability $\mu_{r}\approx 1$. Thermal properties were $\rho_{\mathrm{resin}}\approx 1150\;\mathrm{kg}\;m^{-3}$, $c_{\mathrm{resin}}\approx 1300\;J\;{\mathrm{kg}}^{-1}\;K^{-1}$, and $k_{\mathrm{resin}}\approx 0.25\;W\;m^{-1}\;K^{-1}$. Compared with cortical bone (higher permittivity and finite conductivity) and marrow (conductive, high water content), the resin provides a geometrically realistic but electrically simplified medium. Under the low-frequency magnetic excitation considered here, implant heating is governed primarily by eddy currents induced within the metallic implant; therefore, the surrounding dielectric mismatch has limited influence on implant power deposition but may modestly affect secondary heat diffusion. The resin phantom thus represents a conservative and reproducible environment for implant heating assessment.

The implanted device was a stainless steel rod (medical-grade stainless steel, $\sigma \approx 1.4\times 10^{6}\;S\;m^{-1}$, $\mu_{r}\approx 1$). The implant was positioned within the medullary canal of the tibia phantom across the fracture-site region. The implant axis was approximately aligned with the long axis of the tibia and oriented relative to the excitation coil such that induced circumferential eddy currents could develop along the conductive implant body. Because eddy-current heating depends strongly on implant conductivity, geometry, and orientation with respect to the applied magnetic field, these parameters were fixed and reproduced across all experiments.

The effective specific absorption rate was derived from the initial implant temperature rise using a lumped-capacitance approximation. Neglecting conductive and convective heat losses at early times, the absorbed electromagnetic power satisfies

(17) $$mc\frac{dT}{dt}\approx P_{\mathrm{abs}},$$

which yields

(18) $${\mathrm{SAR}}_{\mathrm{eff}}=\frac{P_{\mathrm{abs}}}{m}\approx c\;\frac{dT}{dt}.$$

The temperature slope dT/dt was obtained from the initial linear segment of the temperature–time curve, during which the implant temperature increased approximately linearly and thermal losses were small relative to absorbed power. This early-time regime corresponds to the lumped-capacitance limit of the bioheat equation, where internal energy storage dominates over conduction and boundary heat transfer.

In the present study, ${\mathrm{SAR}}_{\mathrm{eff}}$ represents a localized effective absorbed power per unit mass associated with the implant region contributing to the measured surface temperature rise, rather than a spatially averaged tissue SAR. The term “effective absorbed power” therefore denotes the net electromagnetic power deposition inferred thermally under lumped conditions.

The sensitivity of ${\mathrm{SAR}}_{\mathrm{eff}}$ to assumed thermal properties follows directly from the lumped-capacitance formulation. Because ${\mathrm{SAR}}_{\mathrm{eff}}=c\;dT/dt$, the estimate depends linearly on the specific heat capacity *c*, whereas density cancels through the mass-normalized definition. For the metallic implant, sensitivity was evaluated by varying the specific heat capacity within representative metallic ranges (c=450–$550\;J\;{\mathrm{kg}}^{-1}\;K^{-1}$), yielding approximately ±10% variation in ${\mathrm{SAR}}_{\mathrm{eff}}$. For the resin phantom, thermal properties representative of photo-polymer materials were assumed ($\rho_{\mathrm{resin}}\approx 1150\;\mathrm{kg}\;m^{-3}$, $c_{\mathrm{resin}}\approx 1300\;J\;{\mathrm{kg}}^{-1}\;K^{-1}$, $k_{\mathrm{resin}}\approx 0.25\;W\;m^{-1}\;K^{-1}$). Variation within typical polymer heat-capacity ranges (c=1200–$1500\;J\;{\mathrm{kg}}^{-1}\;K^{-1}$) would therefore produce approximately ±15% variation in ${\mathrm{SAR}}_{\mathrm{eff}}$.

Based on the results observed in Table 2 and Table 3, the temperature rise in the metallic configuration is consistent with resistive heat generation induced by eddy currents. The higher electrical conductivity of the implant allows larger induced eddy current densities under the applied magnetic excitation. These induced eddy currents generate localized Joule heating, which explains the gradual temperature increase in the implant configuration. Stabilization of the temperature around the 20th minute suggests that heat generation within the implant is balanced by heat dissipation to the surrounding medium. In contrast, the absence of measurable temperature rise in the non-implant configuration indicates that the Joule heating within biological tissue with low conductivity remains minimal under the same excitation. Moreover, the negligible thermal response observed under 0.1 A excitation at 10 kHz further confirms that temperature rise observed in the implant configuration is strongly dependent on the applied current amplitude.

The experimentally inferred specific absorption rate is defined as

(19) $${\mathrm{SAR}}_{\mathrm{eff}}=c\;\frac{dT}{dt},$$

where *c* denotes the specific heat capacity of the material and the derivative is evaluated in the initial linear temperature-rise regime. For the metallic implant, a temperature increase of ΔT≈0.4°C within the first three minutes yields an initial temperature rise rate of $\frac{dT}{dt}\approx 4.44\times 10^{-3}\;K/s$. Using a representative metal heat capacity of $c_{\mathrm{implant}}\approx 500\;J/\left(\mathrm{kg}\cdot K\right)$, the corresponding experimentally inferred SAR becomes ${\mathrm{SAR}}_{\mathrm{eff},\mathrm{implant}}\approx 2.2\;W/\mathrm{kg},$ with an estimated range of 2.0–2.4 W/kg. The implant temperature subsequently stabilizes at approximately 0.4 °C, indicating that thermal equilibrium is reached and that no progressive heating occurs under the applied excitation. In contrast, the resin phantom exhibits a substantially smaller temperature increase of ΔT≈0.05 °C over the same interval, corresponding to ${\mathrm{SAR}}_{\mathrm{eff},\mathrm{resin}}\approx 0.8\;W/\mathrm{kg}.$

For contextual interpretation, commonly cited localized tissue SAR reference levels include 2 W/kg in International Commission on Non-Ionizing Radiation Protection (ICNIRP)-based guidance and 1.6 W/kg in Federal Communications Commission (FCC) recommendations. These values are reported here solely to provide a magnitude reference for tissue exposure and should not be interpreted as directly applicable to metallic implants. Accordingly, SAR is employed in this work as a thermal dosimetry concept to interpret localized energy deposition and implant-related heating mechanisms, whereas formal exposure assessment in the considered stimulation regime is primarily governed by stimulation-related metrics such as induced electric field strength and current density in biological tissue.

**Figure 19:**
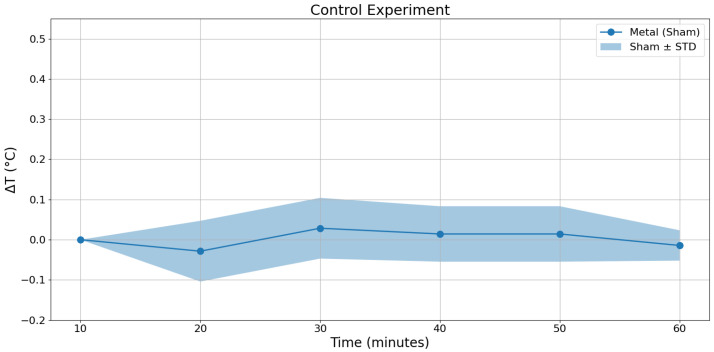
Temperature evolution of the metallic sample under control conditions over 60 min. No electromagnetic excitation is applied.

The temperature evolution under control conditions is presented in Figure 19. The metallic sample does not exhibit any systematic temperature increase during the observation period in the absence of the electromagnetic excitation. The measured temperature fluctuates around 0 °C within ±0.1 °C. The thermal imaging system used to measure temperature values has a minimum temperature resolution of 0.1 °C. Therefore, the fluctuations cannot be interpreted as thermal response of the system.

The absence of monotonic temperature rise confirms that the experimental setup does not introduce artificial heating and that environmental contributions remain negligible.

To quantitatively assess the agreement between the SAR-based thermal model and the experimental measurements, statistical validation metrics were computed for both implant and non-implant configurations under 10 kHz excitation at 0.75 A. The simulated temperature values were sampled at 10 min intervals similar to the experimental measurements. The root mean square error (RMSE), mean absolute error (MAE), maximum absolute error (MaxAE), and Pearson correlation coefficient (*r*) were calculated to evaluate model accuracy. The corresponding equations for these parameters are given below in Table 4.

First, the sample means of the experimental and predicted data are defined as

(20) $$\bar{y}=\frac{1}{N}\sum_{i=1}^{N}y_{i},\;\bar{\hat{y}}=\frac{1}{N}\sum_{i=1}^{N}{\hat{y}}_{i}.$$

RMSE is defined as

(21) $$\mathrm{RMSE}=\sqrt{\frac{1}{N}\sum_{i=1}^{N}{\left(y_{i}-{\hat{y}}_{i}\right)}^{2}}.$$

MAE is given by

(22) $$\mathrm{MAE}=\frac{1}{N}\sum_{i=1}^{N}\left|y_{i}-{\hat{y}}_{i}\right|.$$

MaxAE is defined as

(23) $$\mathrm{MaxAE}=\max_{1\leq i\leq N}\left|y_{i}-{\hat{y}}_{i}\right|.$$

The Pearson correlation coefficient (*r*), which quantifies the linear correlation between the experimental and predicted values, is expressed as

(24) $$r=\frac{\sum_{i=1}^{N}\left(y_{i}-\bar{y}\right)\left({\hat{y}}_{i}-\bar{\hat{y}}\right)}{\sqrt{\sum_{i=1}^{N}{\left(y_{i}-\bar{y}\right)}^{2}}\sqrt{\sum_{i=1}^{N}{\left({\hat{y}}_{i}-\bar{\hat{y}}\right)}^{2}}}.$$

These quantitative validation metrics, as can be seen below in Table 4, demonstrate close agreement between the simulated and experimentally measured temperature evolution. The RMSE was found to be 0.114 °C for the implant configuration and 0.099 °C for the non-implant configuration. Considering that these values are close to the minimum temperature resolution of the thermal imaging system (0.1 °C), the variation between numerical predictions and experimental measurements falls within the sensitivity limits of the thermal imaging device. Therefore, the observed differences remain within measurement uncertainty, suggesting that the SAR-based thermal model provides a reasonable representation of the experimentally observed temperature evolution.

### Internal Peak-to-Surface Temperature Ratio and Sensitivity

Since infrared thermography measures the surface temperature rise $\Delta T_{s}$, we provide a conservative bound on the maximum internal temperature rise $\Delta T_{\max}$ for the tibia phantom. For uniform volumetric heating $\rho \times SAR_{eff}$ in a solid with thermal conductivity *k* and characteristic half-thickness *L*, the maximum internal temperature exceeds the surface temperature by

(25) $$\Delta T_{\max}-\Delta T_{s}\;≲\;\frac{\rho SAR_{eff}L^{2}}{2k}.$$

Defining the peak-to-surface ratio $R_{T}=\Delta T_{\max}/\Delta T_{s}$ gives

(26) $$R_{T}\;\leq \;1+\frac{\rho \;{\mathrm{SAR}}_{\mathrm{eff}}\;L^{2}}{2k\;\Delta T_{s}}.$$

The characteristic half-thickness *L* represents the distance from a potential internal temperature maximum within the tibia wall to the external surface measured by infrared thermography; based on the printed phantom wall thickness in the fracture region, a representative range *L* = 2.5–3 mm was adopted. With ${\mathrm{SAR}}_{\mathrm{eff}}\approx 0.8\;W\;{\mathrm{kg}}^{-1}$ and measured surface rise $\Delta T_{s}\approx 0.1\;{}^{\circ}C$, Equation (26) yields

$$\Delta T_{\max}-\Delta T_{s}\;≲\;0.007–0.017\;{}^{\circ}C,\;R_{T}\;≲\;1.07–1.17.$$

Sensitivity follows directly from Equation (26): $\left(R_{T}-1\right)\propto \left(\rho /k\right)\;{\mathrm{SAR}}_{\mathrm{eff}}\;L^{2}$. Thus, uncertainty is dominated by *L*, while ρ and ${\mathrm{SAR}}_{\mathrm{eff}}$ follow linearly and *k* inversely. Using typical photo-polymer ranges scales the bound proportionally without altering the conclusion that internal peaks remain close to the IR-measured surface rise under the present exposure conditions.

## 5. Discussion

Experimental investigations of thermal effects associated with DEPF-based electromagnetic stimulation remain limited in the literature. Existing PEMF studies mainly focus on biological efficacy or surface temperature measurements in the absence of metallic implants and generally report negligible heating. However, such approaches do not account for implant-induced field perturbations and eddy-current losses, which are inherent to clinically relevant fracture stabilization hardware. The present study addresses this gap by providing controlled experimental evidence, supported by SAR-based dosimetry, that metallic implants can substantially modify local thermal behavior under DEPF excitation.

Electromagnetic stimulation for bone repair has been implemented using PEMF or inductive coupling paradigms, in which relatively uniform low-frequency magnetic fields modulate osteogenic signaling pathways and fracture healing responses; in such systems, any implant-associated temperature rise arises from classical induction and eddy-current dissipation in conductive metallic components rather than from the biological mechanism [16,52]. In contrast, the DEPF framework considered here is intrinsically a field-gradient–driven interaction that employs spatially non-uniform electric fields to generate forces on polarized constituents proportional to (${\nabla |E|}^{2}$), thereby coupling stimulation to force-mediated transport and micro-environmental modulation rather than to thermal energy deposition [33,49]. Under the quasi-static kHz excitation used here, the DEPF objective is governed by spatial non-uniformity, whereas any implant temperature change is expected to be dominated by conventional induced-current/Joule-loss pathways in the metallic implant, similar in principle to the implant heating considerations discussed in the MRI safety literature [53]. Accordingly, the temperature-rise-based ${\mathrm{SAR}}_{\mathrm{eff}}$ analysis is used to quantify implant heating under DEPF-related exposure conditions. Prior PEMF/inductive bone-stimulation studies do not report experimental ${\mathrm{SAR}}_{\mathrm{eff}}$ characterization in an anatomically realistic implanted tibia phantom under spatially non-uniform, DEPF-oriented kHz excitation.

Synthetic anatomical phantoms are widely used in electromagnetic safety and SAR studies because they provide reproducible geometry and controlled dielectric and thermal properties while avoiding the ethical constraints and biological variability associated with real tissue. In the present work, thermal characterization was therefore performed using a 3D-printed anatomical tibia phantom. However, several physiological heat-transfer mechanisms present in vivo are not represented, including blood perfusion, metabolic heat generation, and active thermo-regulation. These processes generally act to dissipate heat and stabilize tissue temperature, thereby reducing temperature elevations compared with static phantom conditions. Therefore, the absence of perfusion-related heat removal in the phantom implies that the reported temperature elevations correspond to a physiologically conservative upper-bound condition relative to perfused bone tissue.

In particular, perfusion-mediated heat transfer can be described using the Pennes bio-heat framework (Equation (2)), in which the perfusion term $\rho_{b}c_{b}\omega_{b}\left(T_{b}-T\right)$ introduces an effective volumetric heat sink proportional to the temperature difference between the local tissue and arterial blood. Using representative cortical bone perfusion values reported in the literature (0.54 kg m^−3^ S^−1^) [54], a first-order bio-heat analysis indicates that physiological perfusion would reduce the steady-state temperature rise compared with non-perfused conditions by introducing this additional volumetric heat-sink term. Metabolic heat generation in bone is small relative to the electromagnetic power deposition under the present exposure regime [55,56] and is therefore not expected to significantly alter the relative heating patterns. Furthermore, active thermo-regulatory mechanisms in vivo, including temperature-dependent vasodilation and systemic heat redistribution, would further enhance convective heat removal and limit local temperature elevation compared with the passive phantom condition [57]. Consequently, the synthetic phantom configuration employed in this study represents a conservative thermal scenario that likely overestimates the in vivo temperature rise. Future work may incorporate perfused biological tissue models or coupled perfusion thermal simulations to more closely reproduce physiological heat transport. These considerations support the physiological relevance of the observed low temperature elevations and suggest an adequate safety margin under the investigated exposure conditions.

The thermal behavior observed in the phantom experiment can be interpreted within the Pennes bio-heat framework under non-perfused conditions. Because the study employed a 3D-printed resin tibia phantom exposed in air, blood perfusion and metabolic heat generation were absent, and heat transfer was governed by conduction with volumetric heating. External heat decrease occurred through convection and radiation at the air-exposed surfaces, while conductive heat transfer through the mechanical supports was limited due to the small contact area. Under these conditions, the measured temperature change reflects the equilibrium between internal volumetric heating and air-side heat dissipation. In vivo, however, blood perfusion provides an additional volumetric heat-sink mechanism that generally accelerates thermal stabilization and reduces temperature elevations relative to non-perfused phantoms.

The experimentally observed thermal responses are consistent with the theoretical understanding of electromagnetic field interactions with conductive materials. The pronounced temperature rise measured in the implant configuration is attributed to eddy currents induced within the metallic implant under time-varying magnetic excitation. These induced currents give rise to resistive losses, leading to localized heating of the implant and its immediate surroundings.

Quantitatively, the metallic implant exhibits a rapid temperature increase of approximately 0.4 °C during the initial phase of exposure. Based on the initial temperature rise rate, the electromagnetic power absorbed by the implant is expressed as an effective mass-normalized heating rate of $SAR_{\mathrm{eff},\mathrm{implant}}\approx 2.2$ W/kg. This value represents a conservative, early-time estimate derived from a lumped thermal balance and characterizes implant- specific power dissipation rather than a mass-averaged tissue SAR used for regulatory compliance. The observed stabilization of temperature indicates that a dynamic equilibrium between electromagnetic power deposition and heat dissipation is rapidly established, preventing progressive or runaway heating.

In contrast, the non-implant configuration exhibits a negligible thermal response. The resin phantom shows a temperature increase of only 0.05 °C over the same interval, corresponding to an effective mass-normalized absorbed power of $SAR_{\mathrm{eff},\mathrm{resin}}\approx 0.8$ W/kg. These small temperature variations remain close to the measurement sensitivity and are consistent with the low electrical conductivity of tissue-mimicking materials, which limits induced current density and electromagnetic energy absorption. This pronounced contrast between implant and non-implant configurations confirms that localized heating is dominated by implant-related electromagnetic interactions rather than bulk tissue effects.

A clear dependence of the thermal response on excitation amplitude is also observed. At higher current amplitudes, the implant configuration exhibits a pronounced transient temperature rise. However, at lower amplitudes, the induced heating remains below the detection threshold for both configurations. This behavior reflects the quadratic dependence of electromagnetic power absorption on field strength and highlights the importance of excitation parameters in governing implant-associated heating.

Spatial temperature distributions were evaluated using infrared thermography, which provides non-contact surface mapping without perturbing the electromagnetic or thermal environment of the implant phantom system. Internal thermometry using thermocouples in time-varying electromagnetic fields can be challenging because conductive sensor leads may experience induced voltages or parasitic heating, potentially biasing temperature readings unless extensive shielding and filtering are employed. Consequently, implant-heating and electromagnetic exposure typically employ non-metallic probes when internal measurements are required. In the present geometry, introducing internal probes would require drilling access channels into the phantom, locally modifying geometry (air gaps or voids), thermal conduction pathways, and electromagnetic boundary conditions near the implant, which could alter field gradients and confound interpretation of gradient-driven exposure. Therefore, the present study focuses on reproducible surface temperature mapping; incorporation of fiber-optic internal probes will be considered in future work.

Although only a stainless steel implant was investigated, eddy-current heating scales with electrical conductivity and characteristic dimension; therefore, stainless steel represents a conservative case compared with lower-conductivity orthopedic alloys such as titanium, suggesting that the present results provide an upper-bound estimate of implant heating under comparable stimulation conditions.

The applied excitation was a PWM waveform with repetition rate f=10 kHz and duty cycle D=50% at $I_{\mathrm{rms}}=0.75\;A$. The predicted magnetic flux density magnitude at the fracture-site ROI was on the order of B≈0.05–0.2 mT, which lies within the sub-millitesla to millitesla range reported for PEMF-based bone-healing stimulation protocols [58,59]. At 10 kHz, the main concern in international guidance is tissue electromagnetic stimulation (induced electric field and current density) rather than RF thermal SAR limits, which are defined for higher frequencies (≳100 kHz) [60,61]. Accordingly, SAR is used here as a thermal dosimetry concept to interpret localized energy deposition and implant-related heating, while formal exposure assessment in this stimulation regime is primarily governed by stimulation-related metrics.

Several limitations of the present work should be acknowledged. The experiments were conducted using ex vivo tissue-mimicking materials, and physiological mechanisms such as blood perfusion, metabolic heat generation, and active thermoregulation were not represented. In vivo, these processes are expected to further enhance heat dissipation, potentially reducing temperature elevations compared to the present measurements. Accordingly, the reported values may be interpreted as conservative estimates of localized implant-associated heating under controlled conditions.

Future investigations should extend this framework to in vivo models, a broader range of implant materials and geometries, and longer exposure durations. Incorporating physiological heat transfer mechanisms and mass-averaged tissue SAR metrics into numerical models would further improve the predictive capability of DEPF-based thermal analyses and support translation toward clinical applications.

Nevertheless, the results demonstrate that metallic implants can significantly influence localized thermal responses under electromagnetic excitation, even when surrounding tissue-like materials exhibit negligible heating. These findings emphasize the need to explicitly account for implant-induced electromagnetic interactions in the design, optimization, and safety assessment of DEPF-based fracture healing therapies and related electromagnetic interventions.

## 6. Conclusions

This study systematically investigated the thermal effects of DEPF exposure in tissue-like samples with and without metallic orthopedic implants by combining experimental measurements with SAR-based theoretical modeling. A clear distinction was observed between implant and non-implant configurations under identical electromagnetic excitation conditions.

The experimental results demonstrate that metallic implants can induce a measurable and reproducible temperature rise up to 0.4 °C when the implant configuration is subjected to 0.75 A at 10 kHz current excitation for one hour. This temperature increase is attributed to eddy currents and associated ohmic losses generated within the conductive implant material. In contrast, the temperature fluctuations in the non-implant configuration remained within the measurement sensitivity (0.1 °C), indicating negligible electromagnetic heating in tibia phantom. Furthermore, the magnitude of the temperature rise was found to depend strongly on the applied current amplitude, highlighting the role of electromagnetic field strength in governing localized thermal behavior.

By explicitly incorporating implant configurations into both experimental and numerical analyses, this work addresses an important gap in the existing DEPF literature, which has predominantly focused on non-implant models. Although the experiments were conducted under ex vivo conditions, the results provide a controlled and conservative assessment of implant-related thermal effects and emphasize the need to account for implant-induced field perturbations when evaluating the thermal safety and optimization of DEPF based bone-healing therapies.

The findings highlight the importance of considering metallic implants in the design, dosimetry, and safety assessment of electromagnetic therapeutic applications and provide a foundation for future in vivo and clinical investigations.

## Figures and Tables

**Table 1:** Additive manufacturing parameters used for the fabrication of the tibia phantom with the Phrozen Mega 8K S MSLA 3D printer.

Parameter	Value
Printing technology	Masked stereolithography (MSLA)
Printer model	Phrozen Mega 8K S
LCD resolution	7680 × 4320 pixels (8K)
XY pixel resolution	∼43 μm
Layer thickness	50 μm
Photopolymer resin	Standard MSLA-compatible resin
Normal layer exposure time	Manufacturer-recommended setting
Bottom layer exposure time	Manufacturer-recommended setting
Number of bottom layers	4–6
Build orientation	Optimized to minimize support contact
Post-processing	IPA cleaning and UV post-curing

**Table 2:** Temperature variation at 10 kHz under continuous excitation for I=0.75 A (10 min sampling, n=10). Values are mean ± SD.

Time (min)	Implant Model ΔT (°C)	Non-Implant Model ΔT (°C)
0	0.00±0.00	0.00±0.00
10	0.37±0.05	−0.15±0.02
20	0.45±0.08	−0.10±0.03
30	0.45±0.08	0.00±0.02
40	0.40±0.12	−0.10±0.03
50	0.37±0.13	−0.10±0.03
60	0.37±0.05	0.08±0.02

**Table 3:** Temperature variation at 10 kHz under continuous excitation for I=0.1 A (10 min sampling, n=10). Values are mean ± SD.

Time (min)	Implant Model ΔT (°C)	Non-Implant Model ΔT (°C)
0	0.00±0.00	0.00±0.00
10	0.05±0.08	0.00±0.02
20	0.07±0.10	0.02±0.02
30	0.02±0.12	0.02±0.02
40	0.00±0.11	0.00±0.02
50	0.05±0.10	0.00±0.02
60	0.03±0.08	−0.03±0.02

**Table 4:** Quantitative validation metrics comparing simulated and thermally measured surface temperature evolution under 0.75 A, 10 kHz excitation.

Metric	Implant	Non-Implant
RMSE (°C)	0.11	0.09
MAE (°C)	0.08	0.07
MaxAE (°C)	0.18	0.15
*r*	0.98	0.93
Peak ΔT (mean ± SD) (°C)	0.45±0.08	0.05±0.02

## Data Availability

The original contributions presented in this study are included in the article. Further inquiries can be directed to the corresponding author.
